# Supplementary material for: Cognitive Considerations in Major Depression: Evaluating the Effects of Pharmacotherapy and ECT on Mood and Executive Control Deficits
Source: Brain Sci. 2022 Mar 4;12(3):350. doi: 10.3390/brainsci12030350 (PMC8946784; doi:10.3390/brainsci12030350)
Supplement: Supplementary file 1 [file brainsci-12-00350-s001.zip › brainsci-1567453-supplementary.pdf]

Supplementary Methods

The Lateralized Attention Test-Revised (LANT-R) consists of a simple computerized task requiring the participant to indicate the direction of an arrow, presented at 6 degrees of the visual field to the right or to the left of a central fixation point, by performing an up or down button press. The target was presented together with two other arrows on each side, which could point either to the same (congruent flanker condition; 144 trials) or to the opposite (incongruent flanker condition; 144 trials) direction. The presentation of the target was preceded by one of three cue conditions: (1) double cue, in which both locations where the target could be presented were highlighted by the blinking of a black box (48 trials); (2) spatial cue, in which only one of the locations where the target could be presented was highlighted by changing the contours of the black box from black to white (192 trials); (3) no cue, in which no changes happened on the screen (48 trials). The spatial cue was designed to validly (144 trials) or invalidly (48 trials) orient the participant's attention to either the left or the right side, thus providing both temporal and spatial information about the impending target. The interval between the appearance of the cue and the presentation of the target was randomized across three intervals (i.e., 0, 400, and 800 ms). The total number of trials was set to 288, with 144 being valid cues, and the remaining trials equally split into double cues, no cues, and invalid cues. The mean trial duration was 5000 ms, the mean block duration was 420 s (84 trials each), and the entire experiment lasted about 30 min (4 blocks total). A short practice block of 32 trials was administered before the beginning of the experimental session, in which participants received visual feedback on accuracy and response time on each trial. See Spagna et al. (2020) for additional information.

Supplementary Results

Additional Results from HAMD ANOVA

From the ANOVA conducted on HAMD scores with the factors *Group* and *Session*, there was strong evidence for a main effect of *Session* ($F(1,93) = 767.02$; $p < .001$; $\eta_p^2 = 0.89$; $BF_{incl} = 1.04 \times 10^{25}$), with pretest scores (median (QR): 21 (7)) being greater than post-test scores (median (IQR): 4 (7); $W = 3190$; $p < .001$; $\hat{r} = 0.92$; $BF_{10} = 5.66 \times 10^4$; $\delta = 1.21$; 95% CI (0.72, 1.51)). There was also strong evidence for a main effect of *Group* ($F(2,93) = 333.12$; $p < .001$; $\eta_p^2 = 0.88$; $BF_{incl} = 1.69 \times 10^{26}$), with the ECT group's scores (median (IQR): 15.5 (4.25)) being greater than the HC group (median (IQR): 1.25 (2.25); $U = 920$; $p < .001$; $\hat{r} = 1.00$; $BF_{10} = 5528.75$; $\delta = 1.44$; 95% CI (0.84, 2.05)) and less than the DT group (median (IQR): 23 (5.5); $U = 657.50$; $p < .001$; $\hat{r} = 0.73$; $BF_{10} = 179.97$; $\delta = -1.05$; 95% CI (−1.64, −0.46)). Furthermore, the DT group's scores were greater than those of the HC group ($U = 1320$; $p < .001$; $\hat{r} = 1.00$; $BF_{10} = 2.00 \times 10^5$; $\delta = 1.54$; 95% CI (1.02, 2.08)).

Additional Results from ANOVA Conducted on the Conflict Effect Estimated on RT

The following presents results from the ANOVA conducted on the conflict effect (CE) estimated on RT with the factors *Group*, *Session*, and *Hemisphere.* There was strong evidence for a main effect of *Session* ($F(1,93) = 20.52$; $p < .001$; $\eta_p^2 = 0.18$; $BF_{incl} = 6.48 \times 10^5$), with pretest CE (median (IQR): 115.5 (56.38) ms) being greater than the post-test CE (median (IQR): 96.5 (33.38) ms; $W = 3222$; $p = .001$; $\hat{r} = 0.38$; $BF_{10} = 55.55$; $\delta = 0.38$; 95% CI (0.17, 0.58)). There was a main effect of *Hemisphere* ($F(1,93) = 4.52$; $p = 0.013$; $\eta_p^2 = 0.09$; $BF_{incl} = 1.045$), with the CE in the LH (median (IQR): 115.25 (46.5) ms) being greater than the CE in the RH (104 (42.5) ms; $W = 3075$; $p = 0.003$; $\hat{r} = 0.35$; $BF_{10} = 23.43$; $\delta = 0.34$; 95% CI (0.15, 0.55)).

There was evidence for the absence of the main effect of *Group* ($F(2,93) = 0.98$; $p = 0.38$; $\eta_p^2 = 0.02$; $BF_{incl} = 0.21$) and for the absence of the following interactions: *Group* × *Hemisphere* interaction ($F(1,93) = 1.24$; $p = 0.29$; $\eta_p^2 = 0.03$; $BF_{incl} = 0.10$), *Session* × *Hemisphere* ($F_{1,93} = 2.16$; $p = 0.15$; $\eta_p^2 = 0.02$; $BF_{incl} = 0.29$), *Group* × *Session* × *Hemisphere* ($F(2,93) = 0.45$; $p = 0.64$; $\eta_p^2 = 0.01$; $BF_{incl} = 0.12$).

Additional Results from Exploratory Hierarchical Clustering Analysis According to the Pretest Conflict Effect Estimated on RT

The following presents results from the ANOVA conducted on HAMD scores with the factors *Cluster* and *Session* separately for the ECT and DT groups. In the ECT group, there was strong

evidence for a main effect of *Session* ($F(1,21) = 105.61$; $p < 0.001$; $\eta_p^2 = 0.83$; $BF_{incl} = 7.56 \times 10^{29}$), with the pretest score (median (IQR): 25 (5.56)) being greater than the post-test score (median (IQR): 92.5 (44.5)). The main effect of *Cluster* did not reach statistical significance ($F(1,21) = 2.33$; $p = 0.14$; $\eta_p^2 = 0.10$; $BF_{incl} = 0.92$). Similarly in the DT group, there was strong evidence for a main effect of *Session* ($F(1,21) = 537.73$; $p <.001$; $\eta_p^2 = 0.95$; $BF_{incl} = 4.68 \times 10^{13}$), with the pretest score (median (QR): 38 (9)) being greater than the post-test score (median (IQR): 8 (5)). There was evidence for the absence of the main effect of *Cluster* ($F(1,21) = 0.05$; $p = 0.96$; $\eta_p^2 = 0.003$; $BF_{incl} = 0.28$).

The following presents results from the ANOVA conducted on the conflict effect (CE) estimated on RT with the factors *Cluster* and *Session* separately for the ECT and DT groups. In the ECT group, there was strong evidence for a main effect of *Session* ($F(1,21) = 52.83$; $p < 0.001$; $\eta_p^2 = 0.72$; $BF_{incl} = 334.29$), with the pretest CE (median (IQR): 131 (63.75) ms) being greater than the post-test CE (median (IQR): 92.5 (39.22) ms). There was also evidence for a main effect of *Cluster* ($F(1,21) = 18.57$; $p < 0.001$; $\eta_p^2 = 0.47$; $BF_{incl} = 217.6$), with the CE of Cluster 1 (median (IQR): 146 (37.13) ms) being greater than the CE of Cluster 2 (median (IQR): 102.75 (50.88) ms). In the DT group, there was inconclusive evidence for a main effect of *Session* ($F_{1,21} = 10.31$; $p = .003$; $\eta_p^2 = .26$; $BF_{incl} = 0.79$), with the pretest CE (median (IQR): 128 (81) ms) being greater than the post-test CE (median (IQR): 95 (48) ms). There was strong evidence for a main effect of *Cluster* ($F(2,21) = 17.42$; $p < 0.001$; $\eta_p^2 = 0.49$; $BF_{incl} = 616.94$). Post hoc comparisons showed that the CE in Cluster 1 (median (IQR): 87.5 (26) ms) was less than Cluster 2 (median (IQR): 116.13 (29.13) ms; $U = 34.5$; $p = 0.007$; $\hat{r} = -0.62$; $BF_{10} = 5.202$; $\delta = -0.84$, 95% CI (−1.66, −0.11)) and less than Cluster 3 (median (IQR): 186.38 (67.25) ms; $U = 2$; $p < 0.001$; $\hat{r} = -0.95$; $BF_{10} = 4.20$; $\delta = -1.01$, 95% CI (−2.15, −0.05)). The CE of Cluster 2 was greater than that of Cluster 3 ($U = 6$; $p = 0.002$; $\hat{r} = -0.86$; $BF_{10} = 4.70$; $\delta = -0.95$, 95% CI (−1.98, −0.06)).

Additional Results from ANOVA Conducted on the Conflict Effect Estimated on ER

From the ANOVA conducted on the CE estimated on ER with the factors *Group*, *Session*, and *Hemisphere*, the main effect of *Session* did not reach statistical significance ($F(1,93) = 2.85$; $p = 0.10$; $\eta_p^2 = 0.03$; $BF_{incl} = 1.25$).

The interaction *Session* × *Hemisphere* reached statistical significance ($F(2,93) = 4.90$; $p = 0.03$; $\eta_p^2 = 0.05$; $BF_{incl} = 0.55$). In the LH, the pretest CE (median (IQR): 2.09 (5.56)%) was greater than the post-test CE (median (IQR): 0.69 (4.16)%;$W = 2423$; $p = 0.05$; $\hat{r} = .24$; $BF_{10} = 1.8$; $\delta = 0.23$; 95% CI (0.04, 0.43)), while there was evidence for an absence of a difference between the pretest CE (median (IQR): 1.39 (6.25)%) and the post-test CE (median (IQR): 2.08 (4.86)%) in the RH ($W = 1841$; $p = 0.90$; $\hat{r} = -0.02$; $BF_{10} = 0.11$; $\delta = 0.002$, 95% CI −0.20, 0.20). The difference between the CE in the LH and the CE in the RH did reach significance at the pretest session ($W = 2456.5$; $p = 0.10$; $\hat{r} = -0.02$; $BF_{10} =0.55$; $\delta = 0.18$, 95% CI (−0.02, 0.39)), and there was evidence for the absence of a difference at the post-test session ($W = 1596$; $p = 0.24$; $\hat{r} = -0.15$; $BF_{10} = 0.30$; $\delta = -0.14$, 95% CI (−0.34, 0.06)).

There was evidence for the absence of the main effect of Hemisphere ($F(1,93) = 0.06$; $p = 0.80$; $\eta_p^2 = 6.90 \times 10^{-4}$; $BF_{incl} = 0.11$) as well as for the interactions *Group* × *Hemisphere* ($F(2,93) = 0.09$; $p = 0.91$; $\eta_p^2 = 0.002$; $BF_{incl} = 0.06$) and *Group* × *Session* × *Hemisphere* ($F(2,93) = 1.53$; $p = 0.22$; $\eta_p^2 = 0.03$; $BF_{incl} = 0.17$).

Supplementary Tables

Supplementary Table S1. Subject by subject breakdown of demographic and clinical information.

**Group**	**Age**	**Education (years)**	**Course of the Disease (months)**	**HAMD Pre**	**HAMD Post**	**Episode**	**Medication**	**Dosage**	**Gender**
ECT + DT	26	15	1	14	7	1	Sertraline	100	2
ECT + DT	28	18	60	25	17	1	Duloxetine	60	2
ECT + DT	22	16	12	14	12	1	Venlafaxine	225	2
ECT + DT	41	8	180	36	12	2	Duloxetine	40	2
ECT + DT	48	5	24	30	5	2	Paroxetine	40	2
ECT + DT	39	8	12	34	3	2	Duloxetine	60	2
ECT + DT	31	15	3	28	4	1	Duloxetine	60	2
ECT + DT	30	16	3	16	11	2	Paroxetine	40	2
ECT + DT	24	15	2	24	2	1	Paroxetine	20	1
ECT + DT	19	14	5	25	8	1	Venlafaxine	175	2
ECT + DT	50	8	48	25	4	2	Paroxetine	40	2
ECT + DT	46	8	240	21	0	2	Paroxetine	50	2
ECT + DT	27	18	48	21	1	2	Duloxetine	40	2
ECT + DT	29	11	240	21	0	2	Paroxetine	40	2
ECT + DT	17	9	12	24	15	1	Sertraline	100	2
ECT + DT	28	8	36	27	1	2	Venlafaxine	225	2
ECT + DT	48	5	12	27	1	1	Paroxetine	50	2
ECT +DT	51	14	1	29	8	1	Duloxetine	60	1
ECT + DT	23	16	12	19	7	2	Paroxetine	40	1
ECT + DT	44	8	9	26	5	1	Duloxetine	60	2
ECT + DT	44	14	1	28	4	1	Paroxetine	50	1
ECT + DT	31	9	1	27	5	1	Paroxetine	40	2
ECT + DT	45	15	324	25	9	2	Duloxetine	60	2

Group	Age	Education (years)	Course of the Disease (months)	HAMD Pre	HAMD Post	Diagnosis	Episode	Medication	Dosage	Gender
DT	48	8	132	35	9	Depression	2	Duloxetine	40	1
DT	41	8	2	37	6	Depression	1	Paroxetine	20	2
DT	45	5	24	32	4	Depression	2	Duloxetine	40	1
DT	57	11	1	35	8	Depression	1	Duloxetine	40	1
DT	20	13	72	41	10	Depression	2	Venlafaxine	225	2
DT	39	16	192	36	5	Depression	2	Duloxetine	60	1
DT	24	16	48	36	6	Depression	2	Sertraline	75	2
DT	43	8	144	41	10	Depression	2	Sertraline	50	2
DT	51	16	216	21	6	Depression	2	Paroxetine	20	2
DT	23	16	120	26	4	Depression	2	Sertraline	50	2

DT	32	16	48	38	4	Depression	2	Paroxetine	30	2
DT	51	8	240	38	10	Depression	2	Duloxetine	60	1
DT	27	12	132	50	6	Depression	2	Sertraline	75	2
DT	23	16	3	33	13	Depression	1	Duloxetine	30	2
DT	23	16	24	41	11	Depression	2	Venlafaxine	150	1
DT	31	16	48	31	5	Depression	2	Duloxetine	60	2
DT	46	8	36	42	9	Depression	1	Duloxetine	60	2
DT	39	16	1	42	10	Depression	1	Paroxetine	30	2
DT	51	8	108	27	6	Depression	2	Paroxetine	20	1
DT	33	11	12	31	8	Depression	1	Duloxetine	60	2
DT	40	9	3	20	1	Depression	1	Citalopram	10	2
DT	39	15	24	44	8	Depression	1	Citalopram	15	2
DT	24	15	1	40	10	Depression	1	Paroxetine	40	2
DT	22	11	48	25	0	Depression	2	Fluoxetine	20	2
DT	49	11	72	44	4	Depression	2	Duloxetine	60	2
DT	18	11	2	42	6	Depression	1	Duloxetine	60	2
DT	34	11	2	48	8	Depression	1	Duloxetine	60	2
DT	26	16	6	36	4	Depression	1	Duloxetine	60	1
DT	18	11	12	38	6	Depression	2	Sertraline	50	2
DT	28	11	156	50	8	Depression	2	Paroxetine	50	2
DT	41	12	60	44	10	Depression	2	Sertraline	100	2
DT	25	9	24	50	11	Depression	2	Duloxetine	40	2
DT	18	12	36	40	10	Depression	2	Venlafaxine	150	2

ECT + DT = electroconvulsive therapy + pharmacotherapy; DT = pharmacotherapy; HAMD = 17-item Hamilton Rating Scale for Depression; gender: 1 = male, 2 = female.

Supplementary Table S2. Measures of central tendency (*median* and *mean*) and of dispersion (*IQR* and *SD*) estimated on response time (RT) and error rate (ER) separately for targets presented in the left hemisphere (LH) or right hemisphere (RH).

			**LH**				**RH**			
			**Congruent**		**Incongruent**		**Congruent**		**Incongruent**	
			Median (IQR)	*Mean* ± *SD*	*Median* (IQR)	*Mean* ± *SD*	*Median* (IQR)	*Mean* ± *SD*	*Median* (IQR)	*Mean* ± *SD*
**Pre**	**HC**	**RT**	720 (180)	747 ± 126	849 (179)	865 ± 133	736 (166)	744 ± 119	8501 (184)	845 ± 202
		**ER**	0.69 (2.08)	1.61 ± 2.45	2.08 (3.65)	2.88 ± 2.77	1.39 (2.78)	1.94 ± 2.20	1.04 (3.65)	2.43 ± 3.70
	**ECT**	**RT**	819 (238)	854 ± 177	971 (206)	996 ± 173	812 (189)	843 ± 176	950 (168)	981 ± 164
		**ER**	2.08 (4.86)	2.87 ± 2.90	4.86 (4.17)	6.22 ± 7.29	0.69 (4.17)	3.29 ± 4.46	2.78 (9.38)	6.61 ± 7.03
	**DT**	**RT**	783 (266)	804 ± 200	971 (328)	941 ± 195	797 (266)	804 ± 202	967 (307)	924 ± 200
		**ER**	2.78 (3.48)	3.03 ± 2.80	9.03 (11.8)	14.04 ± 18.02	2.78 (3.48)	3.09 ± 2.97	6.25 (8.33)	12.14 ± 17.23
**Post**	**HC**	**RT**	708 (132)	715 ± 95	830 (180)	817 ± 104	723 (148)	714 ± 101	819 (147)	816 ± 106
		**ER**	0 (87)	0.78 ± 1.46	1.39 (2.78)	2.03 ± 2.59	0.35 (2.08)	0.90 ± 1.28	2.08 (4.34)	2.46 ± 2.77
	**ECT**	**RT**	872 (198)	887 ± 182	956 (225)	975 ± 164	866 (218)	868 ± 184	945 (245)	958 ± 175
		**ER**	0 (2.78)	3.26 ± 7.21	2.08 (4.87)	5.35 ± 7.83	0 (3.82)	3.17 ± 5.73	2.78 (4.17)	5.61 ± 7.47
	**DT**	**RT**	748 (284)	799 ± 210	952 (247)	914 ± 211	762 (326)	791 ± 210	905 (270)	894 ± 210
		**ER**	0.69 (2.08)	1.47 ± 2.14	4.86 (9.73)	7.26 ± 11.04	2.08 (2.78)	1.98 ± 1.92	5.56 (7.64)	9.24 ± 12.58

Supplementary Table S3. Summary of agglomeration coefficients estimated for clustering analyses of ECT and DT groups indicating suitability of the clustering method.

	**Average**	**Single**	**Complete**	**Ward**
**ECT + DT**	0.94	0.86	0.97	0.98
**DT**	0.94	0.78	0.97	0.98

ECT + DT = electroconvulsive therapy + pharmacotherapy; DT = pharmacotherapy; HAMD = 17-item Hamilton Rating Scale for Depression; gender: 1 = male, 2 = female.

Supplementary Table S4. Descriptive Statistics of HAMD-Scores for the clusters derived from the ECT and DT groups.

		**Pre**		**Post**	
		**Mean ± SD**	**Median (IQR)**	**Mean ± SD**	**Median (IQR)**
**ECT**	Cluster 1	25.86 ± 6.52	27 (3)	8.14 ± 6.59	7 (11)
	Cluster 2	24.06 ± 5.22	25 (6.25)	5.25 ± 3.68	5 (5.25)
**DT**	Cluster 1	38.15± 8.33	38 (9)	7 ± 2.92	8 (5)
	Cluster 2	37.21 ± 6.13	38 (7.75)	6.79 ± 3.17	6 (5)
	Cluster 3	36.17 ± 11.05	37.5 (15)	8.33 ± 3.01	7 (4.25)

Supplementary Table S5. Descriptive statistics of the conflict effect estimated on RT (in ms) at the pre-test (Pre) and post-test (Post) sessions for each cluster in the ECT and DT groups.

		**Pre**		**Post**	
		**Mean ± SD**	**Median (IQR)**	**Mean ± SD**	**Median (IQR)**
**ECT**	Cluster 1	214.29 ± 34.66	214 (53.25)	140.06 ± 61.03	131.00 (63.75)
	Cluster 2	107.59 ± 35.88	120.25 (39.88)	88.87 ± 39.22	92.5 (44.5)
**DT**	Cluster 1	62.62 ± 35.57	74.5 (36)	100.31 ± 49.47	87.5 (43)
	Cluster 2	141 ± 24.25	140.75 (39)	99.79 ± 50.6	90.25 (41.25)
	Cluster 3	240.75 ± 28.6	237.75 (37.63)	149.08 ± 93.19	126.75 (110.75)
